# Novel Insights into Enzymatic Thermostability: The “Short Board” Theory and Zero‐Shot Hamiltonian Model

**DOI:** 10.1002/advs.202402441

**Published:** 2024-09-23

**Authors:** Min Liao, Shihao Feng, Xiaoqing Liu, Guoshun Xu, Sicong Li, Yingguo Bai, Huiying Luo, Bin Yao, Haobo Wang, Tao Tu

**Affiliations:** ^1^ State Key Laboratory of Animal Nutrition and Feeding, Institute of Animal Sciences Chinese Academy of Agricultural Sciences Beijing 100193 China; ^2^ Changping Laboratory Beijing 102200 China; ^3^ Hangzhou Levinthal Biotech Ltd. Zhejiang 311200 China

**Keywords:** coevolution, “short board” theory, thermostabilization, zero‐shot hamiltonian (ZSH) model

## Abstract

Understanding the mechanism underlying thermostabilization in naturally stable enzymes and enhancing the thermostability of unstable enzymes are crucial aspects in enzyme engineering. Despite the development of various engineering methods, there remains substantial scope for improvement. In this study, a novel concept termed as the “short board” theory is proposed, which conceptualizes proteins as barrels with each component representing a jagged board. Notably, optimizing modifications to the shortest board yields optimal enhancements in terms of thermostability performance. To validate this theory, α‐amylase, an industrial bulk enzyme with multiple domains, is employed as a model enzyme. The existence of “short boards” and their impact on thermostability modification are demonstrated at the domain, residue, and atomic levels through experimental confirmation using domain substitution. Furthermore, a novel thermostable design and prediction model called Zero‐Shot Hamiltonian (ZSH) is established and evaluated on α‐amylase. This coevolutionary approach based on thermostability and deep learning exhibits remarkable success exclusively when applied to enzymes with fixed short boards. The integration of the “short board” theory with the ZSH model presents an innovative tool for enhancing enzymatic thermostability.

## Graphical abstract

This study presents the “short board” theory to explain enzyme thermostability, drawing an analogy between proteins and barrels with uneven wood lengths. It proposes that enhancing the shortest board can lead to stability improvements. Validated using of α‐amylase, it employs a novel Zero‐Shot Hamiltonian model that integrates coevolution and deep learning techniques to advance enzymatic engineering.

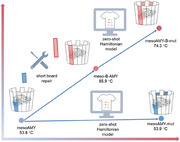

## 1 Introduction

Enzymes, playing a pivotal role as biological catalysts, are indispensable in various life processes and hold significant importance in both industrial and synthetic biology.^[^ ^1^ ^]^ However, their inherent dynamic nature introduces complexities when modulating aspects such as expression, stability, and enzymatic activity.^[^ ^2^ ^]^ With the rapid advancements in deep neural networks, remarkable progress has been achieved in protein design. Cutting‐edge tools for protein structure prediction like AlphaFold^[^ ^3^ ^]^ and RosettaFold,^[^ ^4^ ^]^ along with user‐friendly platforms such as ColabFold,^[^ ^5^ ^]^ have substantially enhanced our comprehension of protein mechanics. These advancements pave the way for innovations in drug discovery and biotechnological applications. In the field of protein design, state‐of‐the‐art techniques such as gradient backpropagation‐based trDesign^[^ ^6^ ^]^ and RFdiffusion,^[^ ^7^ ^]^ as well as graph neural networks like proteinMPNN,^[^ ^8^ ^]^ ESM‐IF,^[^ ^9^ ^]^ and GCN^[^ ^10^ ^]^ design have emerged. Furthermore, sequence‐based models including coevolutionary design tools utilizing the Markov Random Field method^[^ ^11^ ^]^ and pretraining frameworks leveraging large language models like ProGen^[^ ^12^ ^]^ have gained prominence. Despite these technological advances, engineering enzymes with specific dynamic properties remains a significant challenge.

Recent extensive research has underscored the essential integration of rational design, computational design, directed evolution, and machine learning in the development of precise mutation libraries aimed at enhancing enzyme thermostability.^[^ ^13^, ^14^ ^]^ However, accurately predicting the impact of mutations on protein thermostability remains a persistent challenge due to their reliance on the specific unfolding contribution of targeted regions during thermal deactivation. It is noteworthy that while certain strategies may only yield a marginal increase in thermostability (≈3 °C in *T*_m_ values) after multiple mutations,^[^ ^15^ ^]^ significant stability changes can be induced by just a few or even a single mutation.^[^ ^16^ ^]^ For instance, even minor alterations to essential residues have been demonstrated to result in a substantial increase in optimal temperature by 10–15 °C for certain related proteins.^[^ ^17^ ^]^ These residues often represent critical sites of vulnerability that impact protein stability. Notably, extensive investigations into thermolysin consistently identify its N‐terminal surface area as a crucial vulnerability with distinct mutation effects before and after modifications within this region.^[^ ^18^ ^]^

We propose the concept of the “short board” effect within the realm of protein thermostability, drawing an analogy to the shortest plank in a barrel that constrains its overall capacity. This concept posits that targeting critical weak regions or short boards within proteins plays a decisive role in determining the upper threshold of enzymatic stability and holds substantial potential for enhancing enzyme thermostability. Through computational and evolutionary strategies, we illustrate how this strategy facilitates the engineering of thermostable variants through precise mutations. To exemplify this concept, our study focuses on α‐amylase, a prototypical multi‐domain protein. By conducting a comprehensive analysis at multiple levels, including domain, residue, and atom, with regards to the stability of the enzyme's structural network and performing domain‐swapping experiments, we have successfully identified and validated its structural deficiency—the B domain. Addressing this specific vulnerability results in a significant improvement in thermostability and associated functional parameters.

Nevertheless, the challenge of effectively selecting mutation sites after identifying structural weaknesses on a short board remains significant. Traditional computational tools such like PoPMuSiC have had limited success in designing thermostable variants by identifying these weaknesses.^[^ ^19^ ^]^ In response to this limitation, we drew inspiration from evolutionary principles and developed a thermostability design model called Zero‐Shot Hamiltonian (ZSH). The term “Zero‐Shot” originates from deep learning, indicating that our model is capable of predicting thermostability even without being trained on such datasets. Additionally, the term “Hamiltonian” refers to the measure utilized for predicting thermostability within the model.^[^ ^20^ ^]^ This approach integrates evolutionary insights with NLP‐based artificial intelligence. Extensive mutation experiments were conducted to validate our findings and further enhance protein thermostability. Importantly, these mutations primarily demonstrated efficacy in proteins where the “short board” had been addressed, while showing minimal impact on proteins with unaddressed “short boards”. This highlights the critical significance of the “short board” effect and underscores the necessity of prioritizing specific weak areas when identifying beneficial mutations in other regions of the protein.

## 2 Results

### 2.1 Identifying the “Short Board” in α‐Amylase

**Figure 1:**
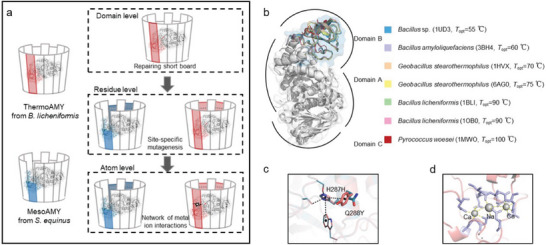
Diagram illustrates the three levels of the “short board” phenomenon. a) Schematic representation of the “short board” phenomenon at the domain, residue, and atomic levels from top to bottom. On the far left of (a), two parental enzymes are depicted: the B domain of a thermophilic α‐amylase thermoAMY derived from *B. licheniformis* (PDB ID: 1BLI) is shown in *pink*, corresponding to the pink board of the barrel. Below, the B domain of a mesophilic α‐amylase mesoAMY from *S. equinus* is displayed in *blue* with its structure predicted by AlphaFold, corresponding to the barrel's blue short board. The top right of (a) refers to chimeric mesoAMY‐B, obtained by replacing the B domain of thermoAMY with that of mesoAMY. The middle right of (a) represents mutations on both meso‐AMY and meso‐AMY‐B to assess the impact of mutations at the same location after repairing this critical domain. The bottom right of (a) indicates absence of metal atoms in the B domain of mesoAMY‐B which does not repair the short board, illustrating enhancement mechanism at atomic level. b) The superimposition of the overall structure of α‐amylase from various *Bacillus* and *Pyrococcus* species reveals significant deviations, particularly in the B domain, as indicated by color‐coding (*blue*, *lightblue*, *wheat*, *paleyellow*, *palegreen*, *pink*, and *red*). c) Enhanced residue‐level interactions are observed after “short board” repair, as indicated by the emergence of novel inter‐residue interactions following the H287H/Q288Y mutation. d) The interaction of metal atoms within the framework of the “short board” phenomenon is crucial, with the Ca‐Na‐Ca triad in domain B, along with its corresponding residue ligands, playing a vital role in mitigating this issue.

α‐Amylase, an extensively studied enzyme renowned for its early production, high yield, and broad industrial applications, has accumulated a substantial amount of multidimensional data covering primary sequence, tertiary structure, and mutation‐related enzymatic properties. Therefore, α‐amylase was selected as an exemplary model for our investigation. In order to investigate the key factors contributing to the stability of thermophilic α‐amylases, a structural alignment was performed on *Pw*AMY (PDB ID: 1MWO), the α‐amylase derived from the hyperthermophilic archaea *Pyrococcus woesei*. This particular α‐amylase is renowned for its exceptional stability and exhibits peak activity at 100 °C. The alignment encompassed several homologs with known structures, including *Bacillus* sp. (PDB ID: 1UD3, optimal temperature *T*_opt_ = 55 °C), *Bacillus amyloliquefaciens* (PDB ID: 3BH4, *T*_opt_ = 60 °C), *Geobacillus stearothermophilus* (PDB ID: 1HVX, *T*_opt_ = 70 °C; PDB ID: 6AG0, *T*_opt_ = 75 °C), and *Bacillus licheniformis* (PDB ID: 1BLI, *T*_opt_ = 90 °C). As shown in **Figure** **1**b, the greatest variance among these diverse α‐amylases is observed in the B domain, which is characterized by an elongated loop inserted within structural domain A. Through the generation and characterization of numerous mutations in α‐amylase, it was revealed that a majority of thermostability‐affecting mutations were localized within the B domain.^[^ ^21^ ^]^ Specifically within this domain, three residues–His152, Cys166, and His168–have been identified as crucial factors contributing to significant differences in thermostability between two α‐amylases differing by 50 amino acids.^[^ ^17^ ^]^ In light of previous indications suggesting its potential vulnerability among enzymes belonging to this class, the B domain was hypothesized to be the most likely “short board”.

**Figure 2:**
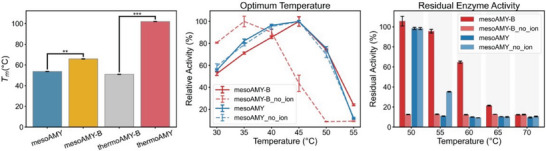
Enzymatic properties of thermoAMY, mesoAMY and their chimeras. a) The *T*_m_ values of each enzyme were determined. b) A comprehensive investigation was conducted to elucidate the influence of Ca^2+^ on the optimal temperature and its impact on enzymatic activity. c) The effect of Ca^2+^ on enzyme functionality was meticulously evaluated by assessing its impact on residual activity at various temperatures. Statistical analysis using *T*‐test confirmed the significance level between *T*_m_ value, where ns indicates *p* > 0.05; ^*^ indicates *p* < 0.05; ^**^ indicates *p* < 0.01; ^***^ indicates *p* < 0.001. The optimal temperature and residual enzyme activity assays were independently performed three times, with data presented as mean ± SD.

To investigate our hypothesis, we selected two enzymes with distinct variations in both sequence and functionality: a thermophilic α‐amylase thermoAMY (PDB ID: 1BLI) derived from *B. licheniformis*, and a mesophilic α‐amylase mesoAMY from *S. equinus* (Figure 1a). Despite sharing highly similar 3D structures, they exhibited only 45.1% sequence identity (Figure S1, Supporting Information). ThermoAMY and mesoAMY displayed optimal temperatures of 90 and 45 °C, respectively. The melting temperature (*T*_m_) values for thermoAMY and mesoAMY were determined to be 102 and 54 °C, respectively (**Figure** **2**a), classifying them as representative thermophilic and mesophilic enzymes. Based on our theory of structural limitations, the determining factor for a structure's maximum capacity lies in its shortest constituent rather than the longest, especially when there are variations in their lengths. In contrast to thermoAMY, which exhibits robust thermostability, it is hypothesized that the B domain of mesoAMY, as one of its shorter components, plays a crucial role in maintaining structural integrity within a construct with suboptimal thermostability. By enhancing these shortened segments within mesoAMY, there is potential to enhance enzymatic thermostability, as suggested by our theory (Figure 1b). Furthermore, our theory emphasizes the importance of addressing concerns within the B domain as an initial step before proceeding with further mutations for optimal effectiveness.

To validate our hypothesis regarding the pivotal role of the B domain in protein structure and its contribution to differences in thermostability between thermophilic and mesophilic enzymes, specifically thermoAMY and mesoAMY, we conducted a swap of the B domains between thermoAMY and mesoAMY. The resulting chimeric enzymes, thermoAMY‐B (a chimera obtained by replacing the B domain of thermoAMY with that of mesoAMY) and mesoAMY‐B (a chimera obtained by replacing the B domain of mesoAMY with that of thermoAMY), were subjected to evaluation for their thermostability using the *T*_m_ value as a key parameter (Figure 2a). As expected, mesoAMY‐B exhibited a significant increase in *T*_m_ value (Δ*T_m_* = 12 °C); however, the *T_m_* of thermoAMY‐B decreased to 51 °C, which was even lower than that of mesoAMY. Our theoretical framework suggests that the “short board” plays a determinative role in establishing the upper threshold of enzymatic stability. It is important to note that enzymatic stability is a multifaceted attribute involving various inter‐domain interactions. Consequently, disrupting certain delicate inter‐domain bonds could potentially reduce stability. Our findings indicate that mesoAMY exhibits slightly higher stability compared to thermoAMY‐B, which supports our proposed hypothesis. Additionally, the incorporation a stable B domain in mesoAMY‐B leads to a significant enhancement of its stability by ≈12 °C. This improvement can be attributed to the critical role of the B domain; however, inter‐domain interactions do not significantly contribute to stabilization.

Both mesoAMY and mesoAMY‐B displayed an optimal temperature at 45 °C (Figure 2b). However, at higher temperatures such as 55 °C, the relative activity‒expressed as the ratio of the enzymatic activity at the given temperature to that at the optimal temperature‒of mesoAMY‐B was significantly enhanced, reaching 24.1%, in contrast to mesoAMY, which exhibited a relative activity of 11.3%. Additionally, compared to mesoAMY, mesoAMY‐B demonstrated significantly higher residual activity within elevated temperatures ranging from 55–70 °C (Figure 2c). The residual activity of mesoAMY after heat treatment at 55 °C was recorded at 35.3%, with a complete loss of activity observed beyond 60 °C. In contrast, exposure to temperatures of 60 and 65 °C resulted in residual activities of ≈64.8% and 21.4%, respectively, for mesoAMY‐B, with complete loss of activity observed only at temperatures exceeding 70 °C. These findings provide evidence supporting the functional role of the B domain in enhancing the thermodynamic stability and kinetic stability of chimeric mesoAMY‐B, thereby validating its significance as a stabilizing factor. Consequently, it can be inferred that the presence of the B domain exerts a substantial influence on the thermodynamic characteristics of these proteins.

### 2.2 Rationalizing the B Domain as the Short Board in α‐Amylase

To explore the fundamental influence of the B domain on thermostability from a molecular architecture perspective, we conducted a comprehensive structural analysis of the crystal structure of thermoAMY (PDB ID: 1BLI). The presence of Ca^2+^ is essential for maintaining the structural integrity of α‐amylase. Violet and Meunier^[^ ^22^ ^]^ have reported that thermoAMY demonstrates complete thermostability in the presence of 5 mm Ca^2+^ at temperatures below 75 °C, but this stability is significantly reduced upon addition of 5 mm EDTA (a metal chelator). Thus, our primary focus was directed toward examining the metal triad consisting of calcium‐sodium‐calcium (Figure 1d).^[^ ^23^ ^]^ To evaluate the contribution of Ca^2+^ ions in preserving the thermostability of the B domain, we assessed the stability profiles of mesoAMY and mesoAMY‐B in the absence of Ca^2+^. Interestingly, while the optimal temperature of mesoAMY remained unaffected by the absence of Ca^2+^, a significant decrease of 10 °C was observed in the optimal temperature of mesoAMY‐B (Figure 2b). Moreover, without Ca^2+^, mesoAMY‐B exhibited a notable reduction in its *T*_m_ value to 49.7 °C, representing a substantial drop of 16.2 °C compared to its state with calcium ions (Tables S1 and S2, Supporting Information). In contrast, the *T*_m_ value for mesoAMY remained relatively stable.

Both mesoAMY and mesoAMY‐B were inactivated when exposed to heat at 55 °C for 5 min in the absence of Ca^2+^ (Figure 2c). It is noteworthy that mesoAMY exhibited superior kinetic stability compared to mesoAMY‐B when subjected to 50 °C. Specifically, under these conditions, mesoAMY retained a significantly higher level of residual activity (99% versus 12% at 50 °C; 35% versus 12% at 55 °C). This observation highlights the pivotal role played by calcium ions in enhancing kinetic stability. The inability of the B domain in mesoAMY to effectively utilize calcium ions positions it as a limiting factor and weak point compromising the overall integrity of the barrel structure (Figure 1d). These findings support our hypothesis regarding the substantial contribution made by the B domain toward enhancing the thermostability of α‐amylase.

### 2.3 Construction of Zero‐Shot Thermostability Design Strategy

Homologous protein sequences serve as empirical evidence of natural evolution, providing valuable insights into protein structure^[^ ^3^, ^4^ ^]^ and phylogenetic relationships.^[^ ^24^ ^]^ To harness evolutionary information for the enhancement of thermostability, our design approach integrates the utilization of a protein language model called multiple sequence alignments (MSA) Transformer^[^ ^25^ ^]^ as the primary module. Trained on extensive protein MSA data, MSA Transformer has the capability to discern interaction patterns between residues crucial for thermostability improvement by recovering corrupted MSAs during training.

**Figure 3:**
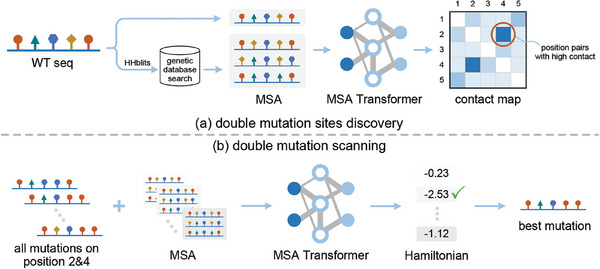
The ZSH model pipeline is focused on identifying double mutations and consists of two stages. a) During the initial stage of site discovery, ZSH utilizes HHblits to retrieve homologous sequences from a genetic database and generates the MSA. Subsequently, a contact map is derived using MSA Transformer based on the generated MSA. The elements within the contact map indicate the probability of two residues being in close proximity within the 3D structure. b) In the mutation scanning stage, ZSH selects the top 1000 contact pairs according to the contact map and performs saturated double mutations. For each mutation, the mutated sequence is input into MSA Transformer along with the MSA generated in stage one. ZSH calculates the Hamiltonian of each mutation using output of MSA Transformer. The optimal mutation is determined by selecting for lowest Hamiltonian value.

**Figure** **3** illustrates the pipeline of our ZSH model, with a primary focus on double mutation. The pipeline is divided into two stages, with the first stage dedicated to identifying double mutation sites. In this stage, it is assumed that double mutations among residue pairs with higher interaction possibility are more likely to enhance thermostability. As shown in Figure 3a, ZSH utilized HHblits,^[^ ^26^ ^]^ a homologous sequence searching tool, to generate the MSA of the wild type α‐amylase. The e‐value and iteration parameters of HHblits were set to 1e^−20^ and 3, respectively. The Uniclust30^[^ ^27^ ^]^ genetic database was employed as the sequence database, resulting in a total of 13549 homologous sequences. After filtering out sequences with gap ratios exceeding 15%, our final MSA consisted of 1601 sequences. Subsequently, we employed the diversity‐maximizing approach outlined in MSA Transformer to meticulously select 64 representative sequences from this pool. These selected sequences, referred to as MSA in Figure 3, were utilized as input for the MSA Transformer model to generate a contact map. The elements within the contact map represent the likelihood of two residues being in close proximity within the 3D structure. Our focus was on identifying potential sites for double mutation by examining the top 1000 contact pairs with the highest probability scores.

The second stage of ZSH involves exhaustive double mutation scanning for each of the 1000 contact pairs. We systematically enumerate all possible double mutations and input each mutated sequence into MSA Transformer, along with the MSA generated in the first stage. The output of MSA Transformer for each mutation contains an *L*×20 matrix *M*, where *L* represents the length of α‐amylase and 20 denotes the 20 natural amino acids. Each vector in *M* contains 20 elements representing the likelihood of each type of amino acid appearing at a specific position in α‐amylase. We postulate that a higher likelihood indicates greater thermostability. By summing the elements corresponding to the residues in the mutated sequence, we derive a measure reflecting the thermostability of the double mutation, referred to as Hamiltonian.

(1) $$Hamiltonian\;=\;-\sum_{i\;=\;1}^{L}\sum_{j\;=\;1}^{20}oh_{ij}M_{ij}$$

Here, *i* and *j* serve as indicators of positions and amino acids, respectively. The one‐hot encoding of the mutated sequence is represented as *oh*, with a dimension of *L*×20. Specifically, vector *oh_i_* contains a value of 1 corresponding to the amino acid at position *i*, while all other elements are set to zero. We performed calculations for all double mutations on 1000 contact pairs and subsequently ranked the mutations based on their Hamiltonian scores. This methodology allowed us to conduct an extensive virtual saturation mutagenesis.

### 2.4 Site‐Specific Mutagenesis After Repairing Short Board

**Figure 4:**
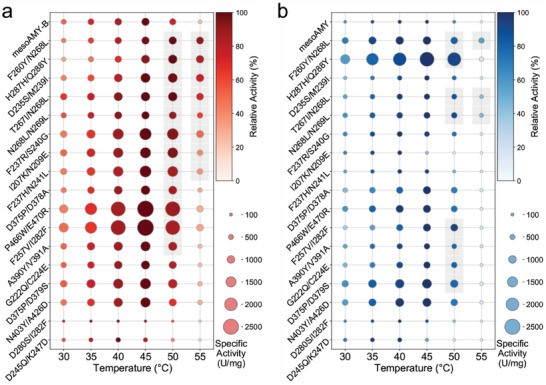
Thermostability and specific activity of mesoAMY, mesoAMY‐B, and its variants. The optimal temperature and specific activity were determined for a) mesoAMY‐B, b) mesoAMY, and their variants. The relative vitality compared to the highest vitality was represented by the intensity of color, while the size of the circle indicated specific vitality.

The double mutations were prioritized based on their Hamiltonian score, and the top 20 predicted variants with the lowest Hamiltonian scores were selected for further experimental validation at corresponding sites within mesoAMY and mesoAMY‐B. These mutation sites are distributed across domains A and C (Figure S2, Supporting Information). Most of these variants exhibited normal expression patterns, with the exception of three (E215P/Y250T, A214G/Y250T, and D245Q/Y250T), which displayed significantly lower expression levels compared to the wild type. The remaining 17 variants underwent additional characterization to determine their temperature optima within the mesophilic range. Among the variants developed using mesoAMY‐B as a scaffold, F260Y/N268L and H287H/Q288Y exhibited a 5 °C increase in their optimum temperature (**Figure** **4**a). In contrast, most variants derived from mesoAMY maintained their optimal temperature, while four variants (I207K/N209E, F237H/N241L, D280S/I282F, and D245Q/K247D) displayed a reduced optimal temperature of 40 °C (Figure 4b). Importantly, in comparison to mesoAMY‐B, eleven mesoAMY‐B variants demonstrated notable enhancements in relative activity at 55 °C. Specifically, variants F260Y/N268L, H287H/Q288Y, D235S/M239I, T267I/N268L, N268L/N269L, and F237R/S240G of mesoAMY‐B displayed relative activities exceeding 50%, while only two variants T267I/N268L and F260Y/N268L of mesoAMY exhibited increased relative activities of 45.9% and 57.2%, respectively, compared to mesoAMY which maintained a peak activity of 13.0%. Moreover, the majority of mesoAMY‐B variants displayed enhanced specific activity with a total of 16 variants showing significantly higher or comparable specific activity to mesoAMY‐B. Particularly notable improvements were observed in variants P466W/E470R and F257V/I282F (Figure 4a). However, corresponding site‐specific mutagenesis in mesoAMY did not result in significant effects (Figure 4b). Additionally, the thermodynamic stability of mesoAMY‐B variants exhibited significantly superior performance compared to the variants of mesoAMY (Figure S3, Supporting Information). These findings provide evidence that implementing thermostability design leads to a more pronounced enhancing effect after addressing its shortcomings.

**Figure 5:**
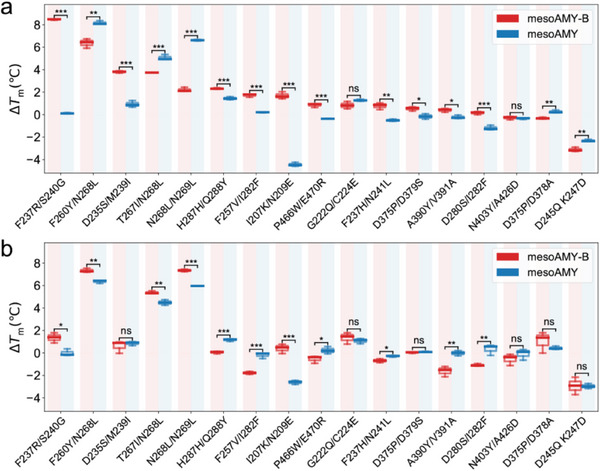
The Δ*T*_m_ values of mesoAMY, mesoAMY‐B, and their variants were compared under two conditions: a) in the presence of exogenously supplemented Ca^2+^ and b) in the absence of exogenously supplemented Ca^2+^. The *T*_m_ value assays were independently conducted three times for each condition. The data were presented as the mean differences between the variants and the wild type ± standard deviation. A *T*‐test was performed to determine if there was a significant difference between the Δ*T*_m_ values, with significance levels indicated as follows: ns, *p* > 0.05; ^*^, *p* < 0.05; ^**^, *p* < 0.01; ^***^, p < 0.001.

Upon the incorporation of Ca^2+^, thermodynamic stability was augmented in mesoAMY‐B variants, as evidenced by Δ*T*_m_ values exceeding 0.5 °C for a subset of 12 variants (**Figure** **5**a; Table S2, Supporting Information). Notably, two variants in particular, F237R/S240G and F260Y/N268L, exhibited substantial elevations in *T*_m_, with increases of 8.5 and 6.4 °C, respectively. Furthermore, the variants D235S/M239I, T267I/N268L, N268L/N269L, and H287H/Q288Y led to temperature increments exceeding 2 °C. In stark contrast, only a minority of six mesoAMY variants showed Δ*T*_m_ values >0.5 °C; however, the observed positive impacts in mesoAMY‐B, such as F257V/I282F, F237H/N241L, I207K/N209E, and F237R/S240G, failed to enhance the corresponding thermostability within the mesoAMY framework. Furthermore, a more pronounced enhancement in both thermodynamic and kinetic stability was observed among the group of variants in mesoAMY‐B compared to mesoAMY when comparing corresponding mutations. Importantly, there was a strong correlation (R^2^ = 0.84) between the changes in *T*_m_ values for these two groups of variants after addressing the limitations present within mesoAMY (Figure S4, Supporting Information). It is noteworthy that in the absence of Ca^2+^ ions, the same mutation had an equivalent effect on the thermostability of both mesoAMY‐B and mesoAMY (Figure 5b; Table S1, Supporting Information), suggesting that targeted mutagenesis becomes increasingly efficacious at augmenting thermostability once the shortcomings inherent to mesoAMY are addressed.

### 2.5 Structural Analysis Reveals the Beneficial Mutations in mesoAMY‐B with Short Board Rectification

**Figure 6:**
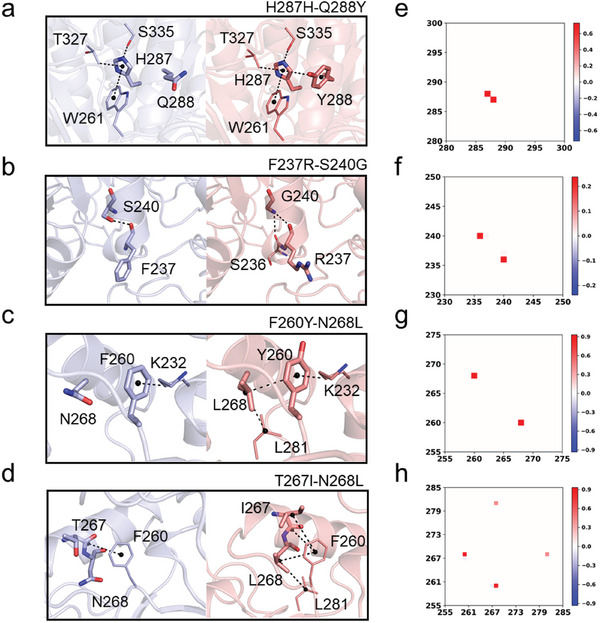
Changes in the interactions among advantageous mutations in mesoAMY‐B and their corresponding contact maps. a,c,e,g) The modifications in interactions between the four variants of mesoAMY‐B before and after mutation were analyzed. The structure of mesoAMY‐B and its variants were predicted by AlphaFold. The wild type was represented by *purple*, while the variants were represented by *salmon*. The contact map signals of each variant b) H287H/Q288Y, d) F237R/S240G, f) F260Y/N268L, and h) T267I/N268L were individually analyzed to gain further insights into their structural alterations.

After rectifying the deficiency, our zero‐shot thermostability design strategy significantly enhanced the thermostability (Figure 1c). To elucidate the underlying mechanisms, we performed a correlation analysis to explore the interaction between enhancements in thermostability, structural modifications, and changes in contact maps within our model. This model provided valuable insights into the conformational dynamics of mesoAMY‐B variants. The mesoAMY‐B variants, particularly F237R/S240G, T267I/N268L, F260Y/N268L, and H287H/Q288Y, exhibited substantial improvements in thermostability through diverse mechanisms. For instance, in variant H287H/Q288Y, the incorporation of a phenolic group onto the Y288 side chain enhanced hydrophobic interactions with the H287 imidazole group (**Figure** **6**a), as evidenced by an intensified signal between residues 287 and 288 on the contact map (Figure 6b). In the case of F237R/S240G, an increase in hydrogen bonding between the C backbone of G240 and S236 within the α‐helix was observed, leading to stabilization of the secondary structure (Figure 6c). This observation is further supported by a modified contact map, which reveals a novel interaction between residues 240 and 236 (Figure 6d). Similarly, the enhanced thermostability observed in F260Y/N268L and T267I/N268L variants can be attributed to the formation of newly established hydrophobic interactions involving L268 and neighboring amino acids (Figure 6e,f), as evidenced by the analysis of contact map (Figure 6g,h). These findings validate the efficacy and precision of our structural‐level thermostability design approach in enzymes where the deficiencies have been rectified.

## 3 Discussion

The limited inherent thermostability of natural proteins often hinders their effectiveness as biocatalysts, biosensors, and in protein therapeutics. The “short board” theory, which draws an analogy between proteins and uneven wooden barrels, suggests that identifying and addressing their weakest points can significantly enhance thermostability, thus establishing a crucial foundation for subsequent site‐specific mutagenesis. In our study, we have identified the B domain—a lengthy loop extending from domain A—as a pivotal weak spot impacting thermostability of α‐amylase. This claim has been supported through meticulous analysis and experimental validation of factors potentially contributing to this vulnerability.

In a broader context, for large proteins with multiple domains, the distal loop region from the catalytic center is often considered a vulnerable point.^[^ ^18^ ^]^ This vulnerability can be attributed to the increased susceptibility of these loop regions to thermal fluctuations and unfolding due to their spatial separation from the core functional sites, ultimately leading to destabilization of the overall protein structure. Addressing this shortcoming in the mesoAMY‐B chimera through domain‐swapping experiments resulted in a remarkable enhancement in thermal resistance and an improvement in related metrics. Conversely, introducing this deficiency into thermoAMY led to a significant decrease in thermostability. These findings highlight the pivotal role of the B domain as a stabilizing element within the protein structure and its potential contribution to observed differences in thermostability between thermophilic and mesophilic enzymes. Indeed, the stability of enzymes, particularly crucial structural elements such as the “short board” discussed in this paper, has been a central focus of research in the field of enzyme engineering. Liu et al.^[^ ^28^ ^]^ employed module substitution, region truncation, and site‐directed mutagenesis to identify the essential structural components associated with the thermostability of β‐mannanase. The restoration of the two “short boards” located at the N‐terminus and C‐terminus of the barrel effectively resulted in a 13.8 °C increase in the *T*_m_ value of the enzyme. Similarly, replenishing “short board” of alcohol dehydrogenase led to a 51 °C increase in its *T*_m_ value.^[^ ^29^ ^]^ These findings, in conjunction with our results, provide evidence for the presence of a “short board” within enzyme proteins and demonstrate that repairing it can effectively enhance enzyme stability.

After addressing the limitations in protein structure, site‐specific mutagenesis strategies have shown a significant improvement in engineering thermostability. It is of utmost importance to possess efficient tools for achieving successful protein design. Machine learning techniques, particularly deep learning approaches, exhibit the potential to extract functional features from extensive protein datasets and provide an unbiased and efficient alternative compared to manual design.^[^ ^14^, ^30^ ^]^ Recent advancements in unsupervised deep learning have further expanded the possibilities of protein design. Tian et al.^[^ ^31^ ^]^ integrated protein co‐evolution with physics principles to abstract the Hamiltonian for adaptive protein design. By decoupling protein co‐evolution from phylogenetic signals and subsequently refining the Hamiltonian, a more targeted approach for designing thermally stable proteins was successfully achieved.^[^ ^32^ ^]^ These evolutionary algorithms inherently incorporate the principle of zero‐shot learning. Leveraging the rapid advancements in deep learning, Rao et al.^[^ ^25^ ^]^ developed MSA transformer that effectively disentangles co‐evolution patterns from phylogenetic signals. Building upon this foundational work, we have reimagined the Hamiltonian and devised the ZSH strategy to provide an accurate characterization of protein thermostability.

Our evolution‐inspired zero‐shot model, which focuses on the structural and evolutionary changes induced by mutations between pairs of residues, has demonstrated remarkable success in enhancing the thermostability of multi‐domain α‐amylases. In the case of mesoAMY‐B, where the “short board” was rectified, ≈70% of the variants exhibited a *T*_m_ value increase exceeding 0.5 °C. The most optimal variant, F237R/S240G, displayed a significant enhancement of 8.5 °C along with other notable improvements. Conversely, in mesoAMY where the “short board” remained unrectified, most mutations had suboptimal effects. These findings strongly indicate that rectifying the “short board” is a foundational prerequisite for effective site‐specific mutagenesis and highlight how evolution‐inspired zero‐shot models are emerging as promising tools for enhancing enzyme thermostability.

In conclusion, our study illuminates the pivotal role of the “short board” in protein engineering by presenting a Zero‐Shot method for enhancing enzyme thermostability, thereby potentially expanding their applications across various biotechnological fields. The pronounced disparity observed between the two datasets underscores the critical role of the “short board” in modulating enzyme thermostability. This discrepancy can be attributed to the inherent propensity of the “short board” region to unfold during protein denaturation, resulting in structural and functional alterations throughout the entire protein molecule. Unlike epistasis and long‐range interactions, which present challenges for comprehensive interpretation, the influence of the “short board” on thermostability is predictable and generally leads to advantageous mutations. However, fully comprehending how these latter factors collectively impact thermostability remains challenging task that does not lend itself straightforwardly to engineering modifications.

## 4 Experimental Section

### Cloning

The coding sequences of two α‐amylases, mesoAMY (GenBank Accession: BAA24178.1) from *S. equinus* and thermoAMY (PDB ID: 1BLI) from *B. licheniformis*, have been previously cloned. Signal peptides were predicted using SingalP 5.0 (http://www.cbs.dtu.dk/services/SignalP/). The mature peptides were synthesized through codon optimization for *Escherichia coli* and subsequently inserted into an expression vector pET‐28a(+) via *Eco*R I and *Not* I restriction sites.

### Chimeras and Variants Construction

The plasmids pET‐28a(+)‐*mesoAMY* and pET‐28a(+)‐*thermoAMY* were utilized as PCR templates for the construction of chimeras. Based on the structural characteristics of mesoAMY and thermoAMY, the enzyme was divided into four domains: a1, b, a2, and c. Specifically, for mesoAMY, these domains correspond to sequences 1‒102, 103‒210, 211‒395, and 395‒486; whereas for thermoAMY they correspond to sequences 1‒100, 101‒206, 207‒393, and 394‒483. Chimeric α‐amylase genes were constructed using polymerase chain reaction using the primers listed in Table S3 (Supporting Information). The chimera mesoAMY‐B was composed of fragments mesoAMY‐a1, thermoAMY‐b, mesoAMY‐a2, and mesoAMY‐c. Chimeric thermoAMY‐B consists of fragments thermoAMY‐a1, mesoAMY‐b, thermoAMY‐a2, and thermoAMY‐c. Subsequently, the resulting DNA fragments were separated by electrophoresis on a 1% agarose gel followed by extraction using the FastPure Gel DNA Extraction Mini Kit (Vazyme, China) and ligation to the vector employing homologous recombinase. Variants of mesoAMY and mesoAMY‐B were generated using protocols outlined in the QuikChange II site‐directed mutagenesis kit (Vazyme, China). The plasmids containing genes for mesoAMY and mesoAMY‐B served as templates for constructing variants. Finally, all constructs were introduced into *E. coli* XL‐10 competent cells through heat shock transformation followed by plasmid extraction after successful sequencing analysis.

### Protein Expression and Purification

The wild type, chimera, and variant plasmids were transformed into the expression host *E. coli* BL21(DE3) for protein production. The transformed cells were cultivated in Luria‐Bertani (LB) medium supplemented with 50 µg mL^−1^ of Kanamycin until reaching an optical density at 600 nm (OD_600_) of 0.8 at a temperature of 37 °C with continuous shaking. Subsequently, isopropyl β‐d‐thiogalactoside (IPTG) was added to a final concentration of 1 mm to induce recombinant protein expression, and cells were incubated at 16 °C for 16 h. Following fermentation, cell harvesting was performed by centrifugation at a speed of 8000 *×g* for 10 min. The resulting cell pellet was resuspended in lysis buffer (20 mm Tris‐HCl, pH7.6) and subjected to ultrasonic disruption for cell lysis purposes. The cell‐free extract was then centrifuged at 12 000 rpm for 15 min to eliminate cellular debris. The resulting supernatant was used for enzyme purification via a Ni‐NTA column equilibrated with three column volumes of equilibration buffer (20 mm Tris‐HCl pH7.6 containing NaCl). The mixed proteins were washed with wash buffer containing imidazole (100 mm imidazole pH7.6 in equilibration buffer), while the target protein was eluted using an elution buffer consisting of high‐concentration imidazole solution (200 mm imidazole pH7.6 in equilibration buffer). Desalting of the purified protein sample was carried out using a Sephadex G‐25 PD‐10 column from GE Healthcare according to the manufacturer's instructions. Subsequently, quality assessment of the purified proteins was conducted through sodium dodecyl sulfate polyacrylamide gel electrophoresis (SDS‐PAGE). Finally, concentration determination on the purified enzyme was accomplished employing bovine serum albumin as standard reference via Bradford method.

### Determination of *T*_m_ Value

To evaluate the thermostability of both the parental proteins and their variants, MicroCal VP‐Capillary differential scanning calorimetry was employed to ascertain their *T*_m_ value. The protein samples were purified and diluted to a concentration of ≈0.5 mg mL^−1^ in a buffer containing 20 mm Tris HCl at pH 7.0. For the temperature scanning process, a range from 25 to 110 °C was selected with a heating rate of 1 °C per minute.

### Analysis of Enzyme Activity

The hydrolytic activity of α‐amylase was determined using the 3,5‐dinitrosalicylic acid (DNS) method. In brief, a diluted purified enzyme (100 µL) was incubated with a substrate containing 1%(w/v) starch in 20 mm phosphate buffer (pH 7.0) at the specified temperature for 30 min. Subsequently, the reaction mixture was boiled for 5 min and rapidly cooled. An aliquot of 250 µL from the reaction mixture was taken to measure the absorbance at 540 nm for detecting released reducing sugars. An enzyme‐free control was included in the analysis. The unit of α‐amylase activity was defined as producing glucose at a rate of 1 µmol per minute under these experimental conditions.

The temperature optima of the purified enzyme was determined by conducting tests in a 20 mm phosphate buffer solution (pH 7.0) across a temperature range of 30 to 55 °C. To assess the thermostability of the enzyme, a diluted enzyme solution (≈100 µg mL^−1^) was preincubated at the designated temperature for a duration of 5 min, followed by immediate cooling on ice. Subsequently, both initial and residual activities were evaluated under standard conditions.

### Statistical Analysis

Three independent experiments were conducted to determine the optimal temperature, specific activity, and residual enzyme activity. Statistical analysis was performed using Prism 8.0 (GraphPad), and the data are presented as mean ± SD. The T‐test was utilized to compare two conditions and determine significant differences, denoted as ns for *p* > 0.05; ^*^, *p* < 0.05; ^**^, *p* < 0.01; ^***^, *p* < 0.001.

## Conflict of Interest

The authors declare no conflict of interest.

## Author Contributions

M.L. and S.F. contributed equally to this work. M.L. contributed significantly to data curation, writing the original draft, and methodology. S.F. was responsible for investigation, methodology, and formal analysis. X.L. played a key role in methodology, resources, and investigation. G.X. handled data curation and investigation. S.L. and Y.B. were involved in investigation, resources, and software development. H.L. also contributed to investigation and resources. B.Y. oversaw the project through supervision, project administration, and funding acquisition. H.W. provided crucial input in conceptualization, methodology, and validation. T.T. was instrumental in conceptualization, writing review and editing, supervision, and funding acquisition.

## Supporting information



Supporting Information

## Data Availability

The data that support the findings of this study are available from the corresponding author upon reasonable request.
