# Supplementary material for: Novel Insights into Enzymatic Thermostability: The “Short Board” Theory and Zero‐Shot Hamiltonian Model
Source: Adv Sci (Weinh). 2024 Sep 23;11(45):2402441. doi: 10.1002/advs.202402441 (PMC11615740; doi:10.1002/advs.202402441)
Supplement: Supplementary file 1 — Supporting Information [file ADVS-11-2402441-s001.docx]

***Supporting Information***

**Novel Insights into Enzymatic Thermostability: The “Short Board” Theory and Zero-shot Hamiltonian Model**

Min Liao, Shihao Feng, Xiaoqing Liu, Guoshun Xu, Sicong Li, Yingguo Bai, Huiying Luo, Bin Yao, Haobo Wang*, Tao Tu*

Author Affiliations:

1. State Key Laboratory of Animal Nutrition and Feeding, Institute of Animal Sciences, Chinese Academy of Agricultural Sciences, Beijing 100193, China

M. Liao, X. Liu, G. Xu, Y. Bai, H. Luo, B. Yao, T. Tu

Email: tutao@caas.cn

2. Changping Laboratory, Beijing 102200, China

S. Feng

3. Hangzhou Levinthal Biotech Ltd., Zhejiang 311200, China

S. Li, H. Wang

Email: haobowang@levinthal.bio

**Table S1. The *T*_m_ values of mesoAMY, mesoAMY-B, and its variants in the absence of added Ca^2+^.**

| Sample | *T*_m_ ℃ | △*T*_m_ ℃ | Sample | *T*_m_ ℃ | △*T*_m_ ℃ |
| --- | --- | --- | --- | --- | --- |
| mesoAMY | 52.98±0.04 | - | mesoAMY-B | 49.74±0.14 | - |
| A390Y/V391A | 52.97±0.22 | -0.01 | A390Y/V391A | 48.12±0.48 | -1.62 |
| T267I/N268L | 57.47±0.23 | 4.49 | T267I/N268L | 55.12±0.14 | 5.38 |
| N268L/N269L | 58.94±0.02 | 5.96 | N268L/N269L | 57.08±0.29 | 7.34 |
| D280S/I282F | 52.32±0.11 | 0.34 | D280S/I282F | 48.66±0.11 | -1.08 |
| G222Q/C224E | 54.06±0.22 | 1.08 | G222Q/C224E | 51.09±0.46 | 1.35 |
| F257V/I282F | 52.79±0.25 | -0.19 | F257V/I282F | 47.96±0.10 | -1.78 |
| D375P/D379S | 53.08±0.04 | 0.1 | D375P/D379S | 49.78±0.03 | 0.04 |
| N403Y/A426D | 52.90±0.48 | -0.08 | N403Y/A426D | 49.24±0.45 | -0.5 |
| P466W/E470R | 53.21±0.26 | 0.23 | P466W/E470R | 49.24±0.26 | -0.5 |
| F237H/N241L | 52.70±0.07 | -0.28 | F237H/N241L | 49.21±0.19 | -0.53 |
| I207K/N209E | 50.38±0.19 | -2.6 | I207K/N209E | 50.15±0.32 | 0.41 |
| D375P/D378A | 53.42±0.17 | 0.44 | D375P/D378A | 50.79±0.83 | 1.05 |
| H287H/Q288Y | 54.14±0.1 | 1.16 | H287H/Q288Y | 49.79±0.06 | 0.05 |
| D235S/M239I | 53.83±0.19 | 0.85 | D235S/M239I | 50.35±0.42 | 0.61 |
| D245Q/K247D | 50.02±0.18 | -2.96 | D245Q/K247D | 46.81±0.74 | -2.93 |
| F260Y/N268L | 59.33±0.14 | 6.35 | F260Y/N268L | 57.07±0.16 | 7.33 |
| F237R/S240G | 52.98±0.31 | 0 | F237R/S240G | 51.10±0.38 | 1.36 |

**Table S2. The *T*_m_ values of mesoAMY, mesoAMY-B, and its variants were determined under the condition of Ca^2+^ addition.**

| Sample | *T*_m_ ℃ | △*T*_m_ ℃ | Sample | *T*_m_ ℃ | △*T*_m_ ℃ |
| --- | --- | --- | --- | --- | --- |
| mesoAMY | 53.75±0.18 | - | mesoAMY-B | 65.86±0.21 | - |
| A390Y/V391A | 53.52±0.16 | -0.01 | A390Y/V391A | 66.26±0.17 | 0.40 |
| T267I/N268L | 58.82±0.24 | 4.49 | T267I/N268L | 69.6±0.01 | 3.74 |
| N268L/N269L | 60.37±0.06 | 5.96 | N268L/N269L | 68.07±0.2 | 2.21 |
| D280S/I282F | 52.57±0.03 | 0.34 | D280S/I282F | 65.01±0.14 | 0.15 |
| G222Q/C224E | 55.02±0.08 | 1.08 | G222Q/C224E | 65.57±0.26 | 0.84 |
| F257V/I282F | 53.95±0.03 | -0.19 | F257V/I282F | 66.57±0.15 | 1.71 |
| D375P/D379S | 53.58±0.24 | 0.1 | D375P/D379S | 66.39±0.18 | 0.53 |
| N403Y/A426D | 53.4±0.06 | -0.08 | N403Y/A426D | 66.58±0.15 | -0.28 |
| P466W/E470R | 53.39±0.03 | 0.23 | P466W/E470R | 66.71±0.18 | 0.85 |
| F237H/N241L | 53.25±0.1 | -0.28 | F237H/N241L | 66.64±0.3 | 0.78 |
| I207K/N209E | 49.3±0.18 | -2.6 | I207K/N209E | 67.55±0.32 | 1.69 |
| D375P/D378A | 54.01±0.13 | 0.44 | D375P/D378A | 65.54±0.08 | -0.32 |
| H287H/Q288Y | 55.2±0.13 | 1.16 | H287H/Q288Y | 68.23±0.25 | 2.37 |
| D235S/M239I | 54.68±0.3 | 0.85 | D235S/M239I | 69.63±0.09 | 3.77 |
| D245Q/K247D | 51.43±0.1 | -2.96 | D245Q/K247D | 61.74±0.2 | -4.12 |
| F260Y/N268L | 61.92±0.16 | 6.35 | F260Y/N268L | 72.23±0.42 | 6.37 |
| F237R/S240G | 53.87±0.05 | 0.12 | F237R/S240G | 74.32±0.05 | 8.46 |

**Table S3. Primers used in this study**.

| Primer name | | sequence (5'→3') | |
| --- | --- | --- | --- |
| thermoAMY-DomainB-F | catattgcgccgctggcggatgtggtgattaaccataaaggcggc | |  |
| thermoAMY-DomainB-R | cgtagatgtttttaatcacttccggatggtcatagtcaatatc | |  |
| mesoAMY-F | cggaagtgattaaaaacatctacgattggg | |  |
| mesoAMY-R | cacatccgccagcggcgcaatatg | |  |
| mesoAMY-DomainB-F | atattaacgtgtatggcgatgtggtgctgaaccataaagcg | |  |
| mesoAMY-DomainB-R | gatgtggcggcggaaattaaacgctggggc | |  |
| thermoAMY-F | gatgtggcggcggaaattaaacgctggggc | |  |
| thermoAMY-R | atcgccatacacgttaatatcgcggctatg | |  |
| mesoAMY-A390Y/V391A-F | gcgccaggcgtatgcatatggaaaagaaatgaac | |  |
| mesoAMY-A390Y/V391A-R | cttttccatatgcatacgcctggcgcaggaacagc | |  |
| mesoAMY-E215P-F | attgggcgccgtggtttgtggaaaccaccg | |  |
| mesoAMY-E215P-R | caaaccacggcgcccaatcgtagatgtttttaatc | |  |
| mesoAMY-Y250T-F | agagaaaaccggcaaagacttttacgtgttc | |  |
| mesoAMY-Y250T-R | ctttgccggttttctctttcatatcgcgg | |  |
| mesoAMY-A214G-F | ctacgattggggtgaatggtttgtggaaacc | |  |
| mesoAMY-A214G-R | caaaccattcaccccaatcgtagatgttttta | |  |
| mesoAMY-N268L/N269L-F | atgagaaaaccctgctggattatctggcgaccaccg | |  |
| mesoAMY-N268L/N269L-R | gccagataatccagcagggttttctcatcgccgttc | |  |
| mesoAMY-D280S/I282F-F | ctttagcctgtttgatgtgcgcctgcatcag | |  |
| mesoAMY-D280S/I282F-R | ggcgcacatcaaacaggctaaagcgatggc | |  |
| mesoAMY-G222Q/C224E-F | aaccacccaggtggaaggctttcgcttagatgcg | |  |
| mesoAMY-G222Q/C224E-R | gaaagccttccacctgggtggtttccacaaaccattc | |  |
| mesoAMY-D245Q/Y250T-F | ctttatccgccagatgaaagagaaaaccgg |  |  |
| mesoAMY-D245Q/Y250T-R | ctctttcatctggcggataaagttgctcatg | |  |
| mesoAMY-F257V- | cttttacgtggttggcgagttttggaacgg | |  |
| mesoAMY-F257V-R | caaaactcgccaaccacgtaaaagtctttgc | |  |
| mesoAMY-I282F-F | tgatctgtttgatgtgcgcctgcatcagaac | |  |
| mesoAMY-I282F-R | gcacatcaaacagatcaaagcgatggccg | |  |
| mesoAMY-D375P/D379S-F | ttgcgcagcagccgtttcaggatagcattgataaactgctgttcctg | |  |
| mesoAMY-D375P/D379S-R | agtttatcaatgctatcctgaaacggctgctgcgcaaattcgccg | |  |
| mesoAMY-N403Y-F | tgataacccgtattgcattggctggagctatc | |  |
| mesoAMY-N403Y-R | agccaatgcaatacgggttatcaaaatagttc | |  |
| mesoAMY-A426D-F | gattaacaacgatcatagcaccgcgaaacgc | |  |
| mesoAMY-A426D-R | cggtgctatgatcgttgttaatcagcaccgc | |  |
| mesoAMY-P466W/E470R-F | cagcttttgggtgggcgcgcggtcagtgagcgcgtatattccgcaggatcag | |  |
| mesoAMY-P466W/E470R-R | ctgcggaatatacgcgctcactgaccgcgcgcccacccaaaagctgcca | |  |
| mesoAMY-F237H/N241L-F | atattgatagccatttcatgagcctgtttatccgcgatatgaaag | |  |
| mesoAMY-F237H/N241L-R | tcgcggataaacaggctcatgaaatggctatcaatatgtttaatcgcat | |  |
| mesoAMY-I207K/N209E-F | tccggaagtgaagaaagaaatctacgattgg | |  |
| mesoAMY-I207K/N209E-R | atcgtagatttctttcttcacttccggatggtcatagtc | |  |
| mesoAMY-D375P/D378A-F | tgcgcagcagccgtttcaggcagatattgataaactgctg | |  |
| mesoAMY-D375P/D378A-R | tatcaatatctgcctgaaacggctgctgcgcaaattcgccg | |  |
| mesoAMY-Q288Y-F | gcctgcattataacctgtttgaagcgagc | |  |
| mesoAMY-Q288Y-R | aacaggttataatgcaggcgcacatcaatc | |  |
| mesoAMY-D235S/M239I-F | taaacatattagcagctttttcattagcaactttatccgcg | |  |
| mesoAMY-D235S/M239I-R | taaagttgctaatgaaaaagctgctaatatgtttaatcgcatctaag | |  |
| mesoAMY-D245Q/K247D-F | ctttatccgccagatggatgagaaatatggcaaagac | |  |
| mesoAMY-D245Q/K247D-R | catatttctcatccatctggcggataaagttgctcatg | |  |
| mesoAMY-F260Y-F | cggcgagtattggaacggcgatgagaaaac | |  |
| mesoAMY-F260Y-R | cttttacgtgttcggcgagtattggaacg | |  |
| mesoAMY-N268L-F | gaaaaccctgaacgattatctggcgaccac | |  |
| mesoAMY-N268L-R | aatcgttcagggttttctcatcgccgttcc | |  |
| mesoAMY-F237R/S240G-F | tattgatagccgtttcatgggtaactttatccgcgatatg | |  |
| mesoAMY-B-A390Y/V391A-F | gcgccaggcgtatgcatatggaaaagaaatgaac | |  |
| mesoAMY-B-A390Y/V391A-R | cttttccatatgcatacgcctggcgcaggaacagc | |  |
| mesoAMY-E215P-F | attgggcgccgtggtttgtggaaaccaccg | |  |
| mesoAMY-E215P-R | caaaccacggcgcccaatcgtagatgtttttaatc | |  |
| mesoAMY-Y250T-F | agagaaaaccggcaaagacttttacgtgttc | |  |
| mesoAMY-Y250T-R | ctttgccggttttctctttcatatcgcgg | |  |
| mesoAMY-A214G-F | ctacgattggggtgaatggtttgtggaaacc | |  |
| mesoAMY-A214G-R | caaaccattcaccccaatcgtagatgttttta | |  |
| mesoAMY-B-N268L/N269L-F | atgagaaaaccctgctggattatctggcgaccaccg | |  |
| mesoAMY-B-N268L/N269L-R | gccagataatccagcagggttttctcatcgccgttc | |  |
| mesoAMY-B-D280S/I282F-F | ctttagcctgtttgatgtgcgcctgcatcag | |  |
| mesoAMY-B-D280S/I282F-R | ggcgcacatcaaacaggctaaagcgatggc | |  |
| mesoAMY-G222Q/C224E-F | aaccacccaggtggaaggctttcgcttagatgcg | |  |
| mesoAMY-G222Q/C224E-R | gaaagccttccacctgggtggtttccacaaaccattc | |  |
| mesoAMY-D245Q/Y250T-F | ctttatccgccagatgaaagagaaaaccgg | |  |
| mesoAMY-D245Q/Y250T-R | ctctttcatctggcggataaagttgctcatg | |  |
| mesoAMY-B-F257V-F | cttttacgtggttggcgagttttggaacgg | |  |
| mesoAMY-B-F257V-R | caaaactcgccaaccacgtaaaagtctttgc | |  |
| mesoAMY-B-I282F-F | tgatctgtttgatgtgcgcctgcatcagaac | |  |
| mesoAMY--BI282F-R | gcacatcaaacagatcaaagcgatggccg | |  |
| mesoAMY-B-D375P/D379S-F | ttgcgcagcagccgtttcaggatagcattgataaactgctgttcctg | |  |
| mesoAMY-B-D375P/D379S-R | agtttatcaatgctatcctgaaacggctgctgcgcaaattcgccg | |  |
| mesoAMY-B-N403Y-F | tgataacccgtattgcattggctggagctatc | |  |
| mesoAMY-B-N403Y-R | agccaatgcaatacgggttatcaaaatagttc | |  |
| mesoAMY-B-A426D-F | gattaacaacgatcatagcaccgcgaaacgc | |  |
| mesoAMY-B-A426D-R | cggtgctatgatcgttgttaatcagcaccgc | |  |
| mesoAMY-B-P466W/E470R-F | cagcttttgggtgggcgcgcggtcagtgagcgcgtatattccgcaggatcag | |  |
| mesoAMY-B-P466W/E470R-R | ctgcggaatatacgcgctcactgaccgcgcgcccacccaaaagctgcca | |  |
| MesoAMY-B-F237H/N241L-F | atattgatagccatttcatgagcctgtttatccgcgatatgaaag | |  |
| MesoAMY-B-F237H/N241L-R | tcgcggataaacaggctcatgaaatggctatcaatatgtttaatcgcatc | |  |
| MesoAMY-B-I207K/N209E-F | tccggaagtgaagaaagaaatctacgattgg | |  |
| MesoAMY-B-I207K/N209E-R | atcgtagatttctttcttcacttccggatggtcatagtc | |  |
| mesoAMY-B-D375P/D378A-F | tgcgcagcagccgtttcaggcagatattgataaactgctg | |  |
| mesoAMY-B-D375P/D378A-R | tatcaatatctgcctgaaacggctgctgcgcaaattcgccg | |  |
| mesoAMY-B-Q288Y-F | gcctgcattataacctgtttgaagcgagc | |  |
| mesoAMY-B-Q288Y-R | aacaggttataatgcaggcgcacatcaatc | |  |
| MesoAMY-B-D235S/M239I-F | taaacatattagcagctttttcattagcaactttatccgcg | |  |
| MesoAMY-B-D235S/M239I-R | taaagttgctaatgaaaaagctgctaatatgtttaatcgcatctaag | |  |
| MesoAMY-B-D245Q/K247D-F | ctttatccgccagatggatgagaaatatggcaaagac | |  |
| MesoAMY-B-D245Q/K247D-R | catatttctcatccatctggcggataaagttgctcatg | |  |
| mesoAMY-B-F260Y-F | cggcgagtattggaacggcgatgagaaaac | |  |
| mesoAMY-B-F260Y-R | cttttacgtgttcggcgagtattggaacg | |  |
| mesoAMY-B-N268L-F | gaaaaccctgaacgattatctggcgaccac | |  |
| mesoAMY-B-N268L-R | aatcgttcagggttttctcatcgccgttcc | |  |
| mesoAMY-B-F237R/S240G-F | tattgatagccgtttcatgggtaactttatccgcgatatg | |  |
| mesoAMY-B-F237R/S240G-R | ggataaagttacccatgaaacggctatcaatatgtttaatc | |  |

**Fig. S1. The alignment of the mesoAMY and thermoAMY sequences (a) and structures (b).** The structure of thermoAMY was retrieved from the Protein Data Bank (PDB ID: 1BLI), while the structure of mesoAMY (GenBank Accession: BAA24178.1) was predicted using AlphaFold.

**
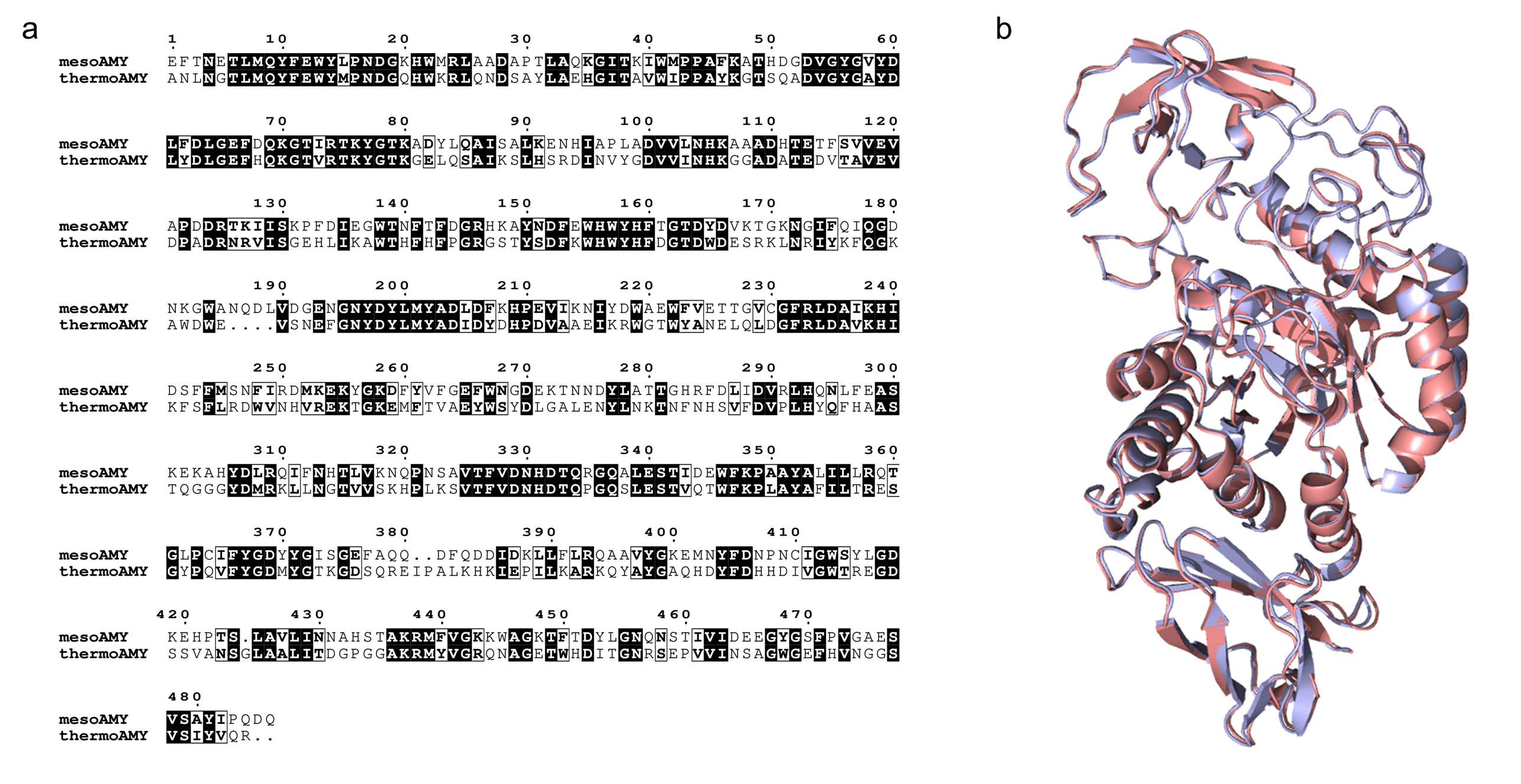
**

**Fig. S2. The locations of mutation sites for 20 predicted variants on the meso-AMY-B sequence.** The meso-AMY-B sequence were utilized in our ZSH model, resulting in the generation of 20 predicted variants with the lowest Hamiltonian. Domain A is represented by *lightblue*, domain B by *silvergray*, and domain C by *orange*.

**
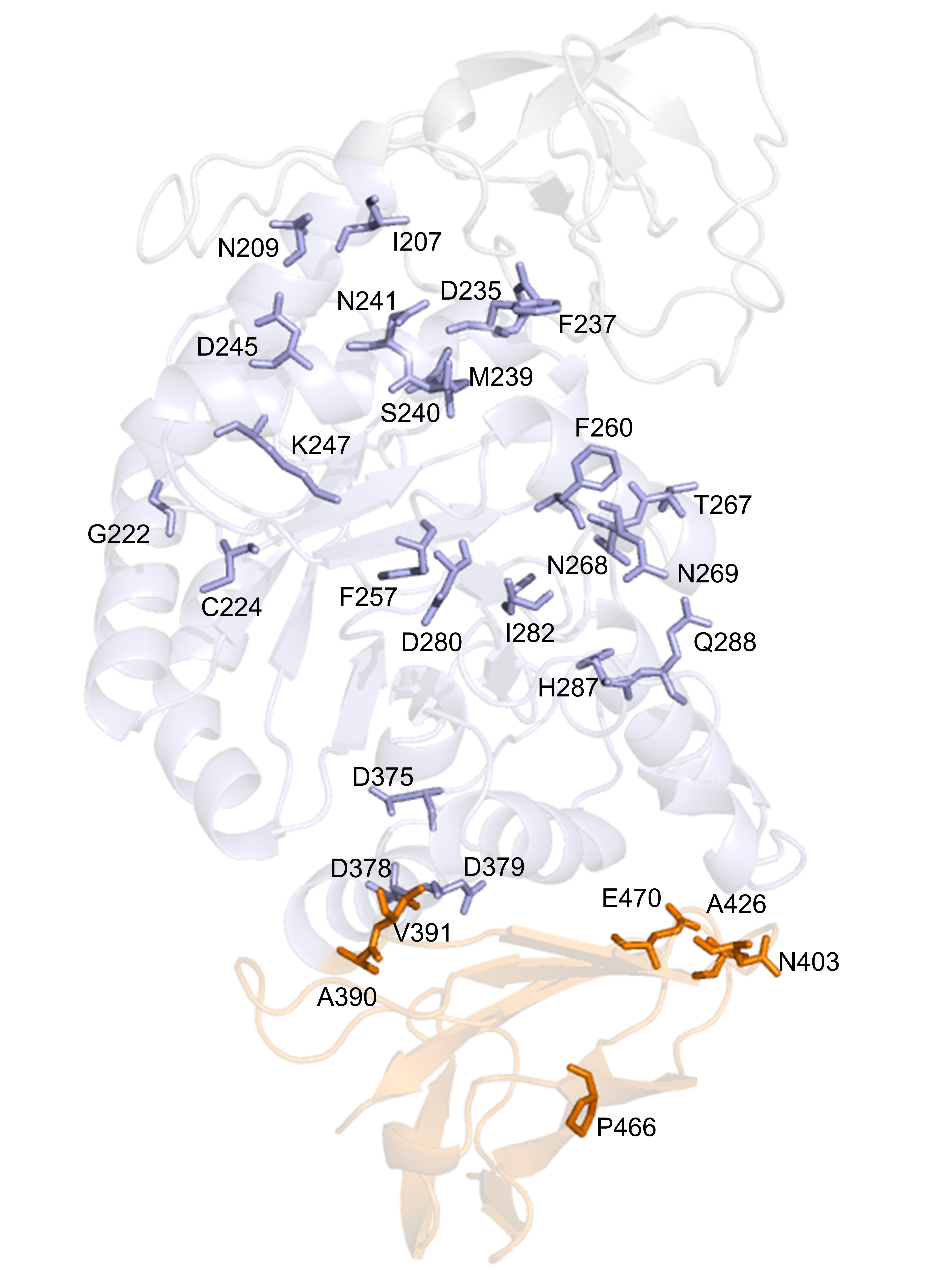
**

**Fig. S3. Thermodynamic stability of mesoAMY, mesoAMY-B and its variants.** The residual enzyme activity of mesoAMY-B and its mutants was quantified following a 5-min thermal incubation at 60 ℃. Similarly, the remaining enzyme activity of mesoAMY and its mutants was determined after subjecting them to a 5-min thermal incubation at 55 ℃.

**

**

**Fig. S4. The correlation between the alterations in *T*_m_ values of mesoAMY-B and mesoAMY was investigated under two conditions: (a) without exogenous Ca^2+^ supplementation, and (b) with exogenous Ca^2+^ supplementation.**

**

**
